# Use of a 5-item modified Fragility Index for risk stratification in patients undergoing surgical management of distal humerus fractures

**DOI:** 10.1016/j.jseint.2021.07.016

**Published:** 2021-09-17

**Authors:** Eliana B. Saltzman, Daniel R. Evans, Albert Anastasio, Ndeye Guisse, Elshaday S. Belay, Oke A. Anakwenze, Mark J. Gage, Tyler S. Pidgeon, Marc J. Richard, David S. Ruch, Christopher S. Klifto

**Affiliations:** a Department of Orthopedic Surgery, Duke University Medical Center, Durham, NC, USA; b Duke University School of Medicine, Durham, NC, USA; c Emory University School of Medicine, Atlanta, GA, USA

**Keywords:** Complications, distal humerus, fractures, frailty, length of stay, readmission

## Abstract

**Introduction:**

We hypothesized that the modified Fragility Index (mFI), which predicts surgical complications, would be applicable to surgical complications in patients older than 50 years with distal humerus fractures (DHF).

**Methods:**

We retrospectively reviewed the American College of Surgeons National Surgery Quality Improvement Program database, including patients older than 50 years who underwent open reduction and internal fixation of a DHF. A 5-item mFI score was calculated. Postoperative complications, readmission and reoperation rates, and length of stay were recorded. Univariate as well as a multivariable statistical analysis was performed, controlling for age, sex, body mass index, length of stay, and operative time.

**Results:**

We identified 864 patients (mean age, 68.6 years ± 10.4), and 74.1% were female. As the mFI increased from 0 to 2 or greater, 30-day readmission rate increased from 3% to 10% (*P* value = .01), rate of discharge to rehabilitation facility increased from 12% to 32% (*P* value = .0), and any complication rate increased from 4% to 19% (*P* value = .0). Rates of pulmonary complications increased significantly in patients with the mFI of 2 or greater (*P* value = .047). Patients with the mFI of 2 or greater were nearly 4 times more likely to be readmitted within 30 days (odds ratio [OR] = 3.5, *P* value = .007) and had an increased OR of 30-day reoperation and any complication (OR = 3.7, *P* value = .02; OR = 4.5, *P* value = .00, respectively) on multivariate analysis.

**Conclusion:**

A fragility state is predictive of postoperative complications, readmission, and reoperation after surgical management of DHF. Our data suggest that a fragility evaluation can help inform surgical decision-making in patients older than 50 years with DHF.

Distal humerus fractures (DHF) comprise 2 to 6% of all fractures and one-third of all humeral fractures, with a bimodal distribution of young, male patients with high-energy mechanisms and elderly, female patients with osteoporosis-related fragility fractures.^13^, ^30^, ^31^ Among patients older than 60 years, there is a projected threefold increase in the incidence of DHF by 2030, a rate that dramatically outpaces the expected growth of this segment of the population.^18^ This highlights the need for specialized fracture care of elderly patients with DHF.

Treatment of DHF can be achieved with either immobilization or surgical fixation. Owing to poor functional outcomes and high rates of malunion, immobilization is typically reserved for low-demand geriatric patients with high perioperative risk profiles.^4^, ^13^ Otherwise, surgical fixation with open reduction and internal fixation (ORIF) remains the treatment of choice with total elbow arthroplasty (TEA)^9^, ^11^, ^13^, ^31^ reserved for highly comminuted fractures with poor bone quality.^23^

However, emerging literature demonstrates favorable outcomes for conservative treatment of DHF in specifically low-demand populations.^1^, ^6^, ^12^ A recent case series reported a nonoperative fracture union rate of 81% with most patients reporting good to excellent subjective outcomes.^12^ Another study evaluated patients with DHF managed nonoperatively and concluded that 95% of this cohort achieved functional range of elbow flexion at 46 months.^1^ Furthermore, there have been a series of comparative studies evaluating the difference in outcomes after ORIF versus TEA for the treatment of DHF. 18, 26, 27, 33 Specifically, a meta-analysis evaluating patients older than 60 years who underwent TEA versus ORIF for DHF revealed similar functional outcome scores and range of motion (ROM), with a trend toward a higher rate of major complications and reoperations after ORIF.^18^ In a prospective randomized control trial, 40 patients older than 65 years with displaced comminuted, intra-articular DHF were randomly assigned either ORIF or TEA. Of the 20 patients assigned to undergo ORIF, 25% were converted to TEA intraoperatively owing to severe comminution and inability to obtain stable fixation, suggesting that TEA may result in decreased reoperation rates.^26^ The mean Mayo Elbow Performance Score was significantly improved in patients who underwent TEA compared with those who underwent ORIF at 3 months, 6 months, 12 months, and 2 years.^26^ Still, TEA comes with its own significant complication profile, limiting widespread implementation.^21^

Given favorable outcomes reported among the various nonoperative and surgical treatment options, it remains unclear which patients should be selected for nonoperative versus surgical management. Because much of the previous literature has been conducted in patients older than 60 years, age alone should not be the metric used in patient selection. Instead, fragility, defined as a generalized decrease in multisystem physiologic reserve and function, should be considered.^40^ Patients of the same age can have greatly different degrees of fragility and, therefore, vastly different operative risk profiles.^7^, ^32^, ^37^

Several studies have used fragility, quantified as the modified Fragility Index (mFI), to effectively predict surgical outcomes and complications in both orthopedics and other surgical specialties.^2^, ^7^, ^16^, ^17^, ^24^, ^29^, ^34^, ^35^, ^39^, ^40^ The mFI is an 11-item index of functional status and comorbidities originally developed as a simplified version of the Canadian Study of Health and Aging Frailty Index, a comprehensive 70-item scale designed to quantify frailty.^37^ The mFI has been compared well with other fragility indices, specifically the Charlson Comorbidity Index, in other areas of orthopedics and general surgery.^5^, ^14^, ^28^ A subsequent abbreviated 5-item index was recently validated against the 11-item mFI score and has been used to successfully predict complications to allow preoperative risk stratification.^36^, ^39^, ^41^ A 2018 retrospective review of 6494 patients, older than 50 years, who had undergone ORIF for distal radius fracture revealed that patients with an mFI of 2 or greater were nearly 2.5 times more likely to incur a postoperative complication.^39^ A larger cohort study encompassing orthopedic, vascular, and general surgery revealed a linear dose-dependent relationship between mFI and 30-day unplanned readmission, postdischarge emergency department visits, predischarge and postdischarge complications, and postdischarge mortality.^38^ These studies underscore the utility of the mFI in the surgical management of these patients.

Assessing patient risk factors and comorbidities by using fragility measures has demonstrated a promising role in the patient selection process to ultimately minimize readmissions, reoperations, and overall complications. We hypothesized that the mFI, which predicts complications after orthopedic surgeries, would predict surgical complications in patients older than 50 years with DHF.

## Material & methods

### Data collection

Data for this study were collected from the American College of Surgeons National Surgery Quality Improvement Program (ACS-NSQIP) database. The NSQIP database is an international, prospective database that collects preoperative and 30-day outcome data for patients undergoing surgical operations across multiple subspecialities. The database captures 95% of 30-day outcomes by observing in-hospital morbidity and mortality and then confirming 30-day outcomes by contacting patients via writing and phone call at the end of the period. In addition, surgical clinical reviewers and random audits ensure the accurate collection of data.

In the present study, the NSQIP database was queried for patients based on Current Procedural Terminology codes. The following codes were used: 24545 (open treatment of humeral supracondylar [not intercondylar] fracture, ± internal/external fixation), 24546 (open treatment of humeral supracondylar or intercondylar fracture, ± internal/external fixation), 24575 (open treatment of humeral epicondylar fracture, medial or lateral, ± internal/external fixation), 24579 (open treatment of humeral condyle fracture, medial or lateral), 24586 (open treatment of periarticular fracture/dislocation elbow), 24587 (open treatment of periarticular fracture/dislocation with implant arthroplasty), and 24615 (open treatment of acute or chronic dislocation). All patients from 2014 to 2017 were initially included in this study. Patients younger than 50 years and those with open injuries were excluded. Additionally, patients who had another surgery were excluded as a proxy to capture only patients with isolated injuries. Finally, patients meeting sepsis or presepsis criteria before surgery were excluded, as were patients with incomplete data available for analysis.

### Patient demographics

Patient demographic data were collected and included the following information: sex, age, race, American Society of Anesthesiologists (ASA) classification, body mass index (BMI) kg/m^2^, and wound classification.

### Modified Fragility Index

The 5-item mFI used in this study included the following 5 patient history items: history of diabetes mellitus, congestive heart failure (CHF) new diagnosis or exacerbation of chronic CHF within 30 days of surgery, hypertension (HTN) requiring medication, history of chronic obstruction pulmonary disease or pneumonia, and nonindependent functional status (partially or completely dependent in activities of daily living within the last 30 days before surgery). The 5-item-mFI score was calculated for every patient by simply adding the number of variables present in patients with a possible score from 0 to 5 (Table I).

**Table 1:** Patient demographics and modified Fragility Index score.

	Total		mFI score						*P* value
			0		1		2+		
Age, median (IQR)	66	59-74	62	57-71	71	63-80	71	63-78	
50-59	198	23.4%	112	37.0%	56	15.6%	30	16.0%	.000
60-69	274	32.3%	107	35.3%	109	30.4%	58	31.0%	
70-79	203	23.9%	48	15.8%	96	26.8%	59	31.6%	
80-89	173	20.4%	36	11.9%	97	27.1%	40	21.4%	
Female sex	626	73.8%	223	73.6%	267	74.6%	136	72.7%	.891
BMI (kg/m^2^)									
Underweight	18	2.2%	7	2.4%	7	2.0%	4	2.2%	.000
Normal weight	229	28.0%	106	36.4%	93	26.7%	30	16.7%	
Overweight	250	30.5%	93	32.0%	104	29.9%	53	29.4%	
Obese	172	21.0%	54	18.6%	82	23.6%	36	20.0%	
Severely obese	78	9.5%	19	6.5%	35	10.1%	24	13.3%	
Morbidly obese	72	8.8%	12	4.1%	27	7.8%	33	18.3%	
Race									
White	639	75.4%	214	70.6%	275	76.8%	150	80.2%	.058
Black	35	4.1%	10	3.3%	18	5.0%	7	3.7%	
Asian	25	3.0%	10	3.3%	8	2.2%	7	3.7%	
Other/unknown	149	17.6%	69	22.8%	57	15.9%	23	12.3%	
ASA score									
Healthy	33	3.9%	33	10.9%	0	0.0%	0	0.0%	.000
Mild	335	39.6%	177	58.4%	135	37.7%	23	12.4%	
Severe	427	50.4%	86	28.4%	201	56.1%	140	75.3%	
Life threat	52	6.1%	7	2.3%	22	6.1%	23	12.4%	
Wound categorization									
Clean	777	91.6%	282	93.1%	327	91.3%	168	89.8%	.755
Clean/contaminated	41	4.8%	11	3.6%	20	5.6%	10	5.3%	
Contaminated	19	2.2%	6	2.0%	8	2.2%	5	2.7%	
Dirty/infected	11	1.3%	4	1.3%	3	0.8%	4	2.1%	
Smoking status									
No	730	86.1%	252	83.2%	327	91.3%	151	80.7%	.001
Yes	118	13.9%	51	16.8%	31	8.7%	36	19.3%	
LOS, median (IQR)	2	1-4	1	0-3	2	1-4	3	1-5	.235
Operative time, median (IQR)	134	88-184	131	87-177	135	90-187	139	85-198	.136
Total	848	100.0%	303	35.7%	358	42.2%	187	22.1%	

*mFI,* modified Fragility Index; *IQR,* interquartile range; *BMI,* body mass index; *ASA,* American Society of Anesthesiologists Classification; *LOS,* length of stay.

### Outcome and complication data

The 30-day outcome data were collected for each patient. Complications were classified into the following broad categories: wound (wound dehiscence or other complications, not including surgical site infection), cardiac (cardiac arrest or myocardial infarction), pulmonary (pneumonia, pulmonary embolism, unplanned reintubation), hematology (deep vein thromboembolism, need for transfusion), renal (progressive renal insufficiency, acute kidney failure), and adverse hospital discharge (discharge to other than home). In addition, data for all complications were analyzed as Clavien-Dindo IV complications, which are those that are life-threatening and cause end-organ dysfunction. Clavien-Dindo IV complications included cardiac arrest, myocardial infarction, septic shock, pulmonary embolism, and renal failure. Both 30-day readmission and reoperation data were also collected and analyzed.

### Statistical analysis

Initial statistical comparison of demographic variables and mFI score was performed with the Kruskal-Wallis test for categorical independent variables and simple logistic regressions for continuous independent variables. To assess for confounders, a bivariate analysis of the association of demographic variables to outcome and complications was performed with a logistic model for continuous independent variables and chi-square for categorical independent variables. Age, BMI, race, smoking status, and length of stay were identified as possible confounders. To assess for association between mFI and each complication and outcome, a bivariate analysis was performed using a logistic model. A subanalysis comparing each mFI component and each outcome was also performed using a univariate and multivariate logistic model. This association was then further examined using a multivariate logistic regression control for potential confounders. A *P* value of less than .05 was considered statistically significant. Statistical analysis was performed with Stata (version 16; StataCorp, College Station, TX, USA).

## Results

### Patient demographics

Eight hundred sixty-four patients who were 50 years old or older and underwent operative management of a DHF were identified from the NSQIP database. Of these, 848 patients had complete data for analysis. The majority of the identified patients were women (73.8%), and the median patient age was 66 years (interquartile range: 60-77). Included patients were predominantly Caucasian (75.4%). Most patients either had a normal BMI (18.5-24.9; 28.0%) or were overweight (BMI: 25-20.9; 30.5%). Nearly all identified patients had an ASA class of 3 or less 93.9%, with the majority being ASA class 3 (50.4%).

### 5-Item mFI scores

The calculated Frailty Index for all patients in the study sample ranged from 0 to 5. However, for statistical purposes, these are reported in the following groups: 0, 1, and 2 or greater. The number of patients with each mFI level was as follows: mFI = 0 in 303 patients (35.7%); mFI = 1 in 358 patients (42.2%); mFI = 2 in 161 patients (19.0%); mFI = 3 in 23 patients (2.7%); mFI = 4 in 3 patients (0.4%); and mF1 = 5 in 0 patients. When combined, the number of patients with mFI score of 2 or greater was 187 (22.1%).

### 30-Day postoperative complications and mFI

An increasing mFI score was significantly associated with an increased risk of readmission (odds ratio [OR]: 1.63 [1.15-2.31], *P* = .006), reoperation (OR: 1.66 [1.08-2.56], *P* = .02), adverse hospital discharge (OR: 1.76 [1.44-2.16], *P* < .001), and any complication (OR: 2.03 [1.57-2.63], *P* < .001) (Table II). Of the complications, a higher mFI was associated with a significantly higher rate of infection (OR: 2.18 [1.14-4.19], *P* = .02) and hematological (OR: 16.97 [2.30-125.22], *P* = .005) complications.

**Table 2:** 30-Day complications by modified Fragility Index scores (univariate analysis).

	Total		0		1		2+		*P* value	OR	*P* value	95% CI
Readmission	41	4.8%	9	3.0%	14	3.9%	18	9.6%	**.002**	1.63	**.006**	1.15-2.31
Reoperation	26	3.1%	6	2.0%	9	2.5%	11	5.9%	**.038**	1.66	**.021**	1.08-2.56
Mortality	7	0.8%	1	0.3%	4	1.1%	2	1.1%	.492	1.67	.214	0.74-3.75
Adverse hospital discharge	170	20.0%	37	12.2%	73	20.4%	60	32.1 %	**.000**	1.76	**.000**	1.44-2.16
Any complication	87	10.3%	13	4.3%	38	10.6%	36	19.3%	**.000**	2.13	.15	0.76-5.92
Clavien-Dindo 4	4	0.5%	0	0.0%	2	0.6%	2	1.1%	.233	2.03	**.000**	1.57-2.63
Total	848		30		35		18					

*OR,* odds ratio; *CI,* confidence interval.

Significant *P* values are indicated in bold.

### 30-Day postoperative outcomes and mFI components

**Figure 1:**
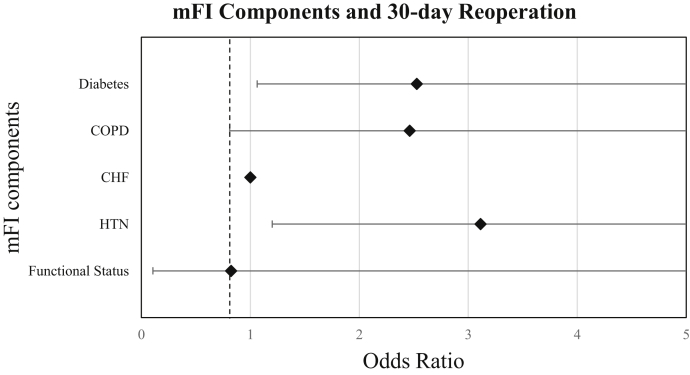
Forest plot of the modified Fragility Index Score and 30-day hospital reoperation. *mFI,* modified Fragility Index.

**Figure 2:**
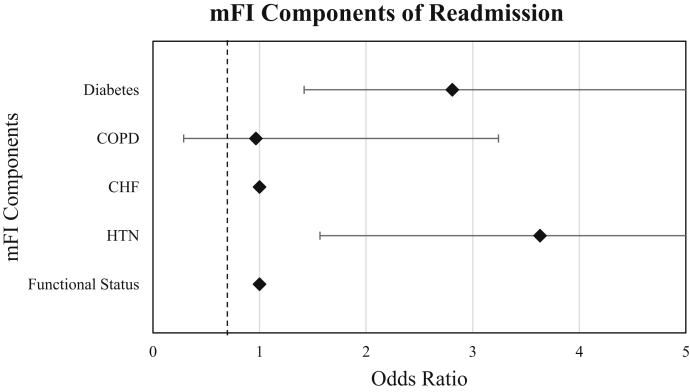
Forest plot of the modified Fragility Index score and 30-day hospital readmission. *mFI,* modified Fragility Index; *COPD,* chronic obstruction pulmonary disease; *HTN,* hypertension; *CHF,* congestive heart failure.

**Figure 3:**
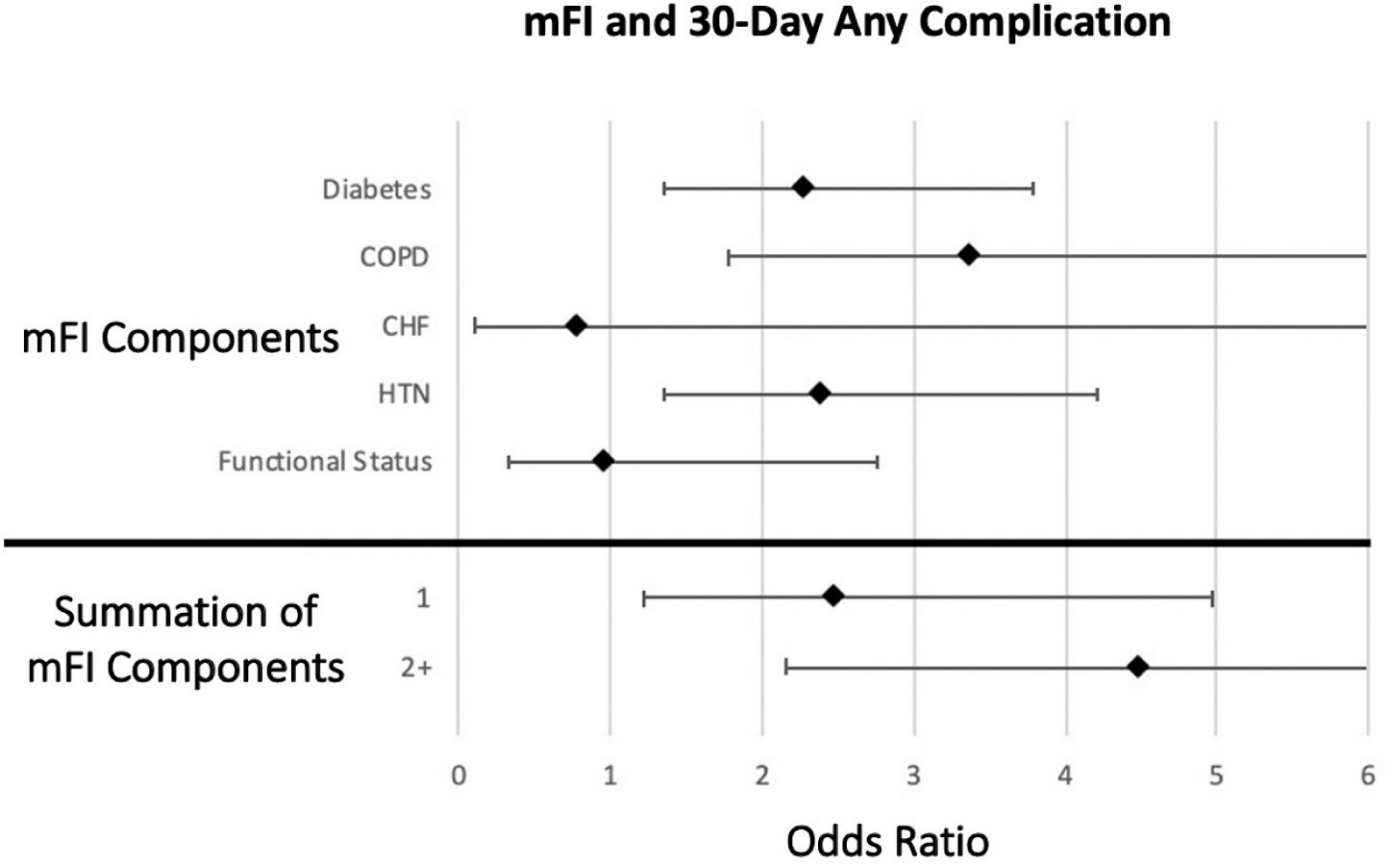
Forest plot of the modified Fragility Index score and 30-day any complication. *mFI,* modified Fragility Index; *COPD,* chronic obstruction pulmonary disease; *HTN,* hypertension; *CHF,* congestive heart failure.

Table III portrays the association between the five components of the mFI score and 30-day postoperative complications. On univariate analysis, diabetes and hypertension were independently associated with higher rates of readmission. All of the mFI variables were independently associated with higher rates of adverse hospital discharge with the exception of CHF. Each of the mFI variables was independently associated with higher rates of any complications with the exception of CHF and functional status. Nonindependent functional status was independently associated with increased mortality. On multivariate analysis, higher rates of readmission and reoperation were associated with CHF, diabetes, and hypertension (Figure 1, Figure 2). Higher rates of adverse discharge were associated with patients who had nonfunctional status. Furthermore, patients with diabetes, chronic obstruction pulmonary disease, and hypertension had an increased risk of any complication (Fig. 3).

**Table 3:** Outcomes vs modified Fragility Index score components (multivariate analysis).

	Readmission			Reoperation			Mortality		
	OR	*P* value	95% CI	OR	*P* value	95% CI	OR	*P* value	95% CI
Diabetes	2.81	**.003**	1.42-5.56	2.53	**.036**	1.06-6.00	0.92	.939	0.10-8.21
COPD	0.97	.956	0.29-3.24	2.46	.112	0.81-7.48	1.74	.616	0.12-15.18
CHF	1.00	**.000**	0.00-0.00	1	**.00**	0.00-0.00	1.00	**.000**	0.00-0.000
Hypertension	3.63	**.003**	1.57-8.42	3.11	**.019**	1.20-8.07	1.61	.601	0.27-9.56
Functional status	1.00	**.000**	0.00-0.00	0.82	.854	0.12-6.41	4.75	.095	0.76-29.55
	Adverse discharge			Any complication					
	OR	*P* value	95% CI	OR	*P* value	95% CI			
Diabetes	1.62	.057	0.99-2.69	2.26	**.002**	1.35-3.79			
COPD	1.30	.462	0.64-2.63	3.36	**.000**	1.78-6.32			
CHF	0.90	.896	0.20-4.08	0.78	.818	0.09-6.48			
Hypertension	1.22	.409	0.76-1.95	2.39	**.003**	1.35-4.21			
Functional status	4.29	**.000**	2.01-9.17	0.94	.913	0.32-2.77			

*OR,* odds ratio; *COPD,* chronic obstructive lung disease; *CHF,* chronic heart failure; *CI,* confidence interval.

Significant *P* values are indicated in bold.

### 30-Day reoperation, readmission, and any complication and mFI

Table IV portrays the results of the multivariate analysis when controlling for age, sex, BMI, race, smoking status, LOS, and operative time. After controlling for these variables, patients with an mFI score of 2 or greater had an increased rate of readmission (OR: 3.51 [1.41-8.73], *P* = .007), reoperation (OR: 3.68 [1.23-11.02], *P* = .020), any complication (OR: 4.48 [2.15-9.29], *P* < .001), and adverse hospital discharge (OR: 2.26 [1.26-4.07], *P* = .006).

**Table 4:** 30-Day complications by modified Fragility Index scores (multivariate analysis).

mFI	Readmission			Reoperation			Mortality			Any complication			Discharge		
	OR	*P* value	95% CI	OR	*P* value	95% CI	OR	*P* value	95% CI	OR	*P* value	95% CI	OR	*P* value	95% CI
**1**	1.66	.28	0.66 -4.2	1.77	.31	0.58 -5.36	2.29	.49	0.22 -23.5	2.46	.01	1.22 -4.97	1.24	.44	0.72 2.14
**2+**	3.51	**.01**	1.41 -8.73	3.68	**.02**	1.23 -11.02	2.72	.44	0.21 -34.45	4.48	**0**	2.15 -9.29	2.26	**.01**	1.26 -4.07

*OR,* odds ratio; *CI,* confidence interval.

Significant *P* values are indicated in bold.

## Discussion

Continued debate remains surrounding the appropriate surgical management of DHF in elderly patients. We present the utility of applying the mFI among patients older than 50 years with DHF in predicting 30-day surgical complications. In a cohort of nearly 900 patients, we concluded that an increase in mFI resulting in increased rates of readmission, discharge to rehabilitation facilities, and rates of any complication. Compared with patients with an mFI of 0, patients with an mFI of 2 or greater were nearly 4 times more likely to be readmitted. It is, to our knowledge, the first analysis of the utility of the mFI applied to the DHF patient cohort.

Broadly defined, two categories of postoperative adverse outcomes should be considered: (1) surgical procedure–specific complications such as need for reoperation or surgical site infection and (2) general medical complications such as development of deep venous thrombosis or adverse cardiac event. Consideration of surgical procedure–specific complications plays a larger role in determining whether or not to pursue operative intervention. However, general medical complications can also have impact on patient well-being and are important factors in treatment planning. Patients with a variety of comorbidities and markers of fragility are at a higher likelihood of developing an adverse outcome from either category.^11^, ^19^ A multicenter study evaluating the Frailty Phenotype (FP) and Frailty Index (FI) scores indicated worse outcomes across a variety of elective orthopedic surgical procedures in patients with high frailty status.^10^ Additionally, the FRAIL scale, which is a short 5-question assessment of fatigue, resistance, aerobic capacity, illnesses, and loss of weight, was predictive of poor outcomes in a geriatric fracture population.^19^

This investigation confirmed our hypothesis that the 5-item mFI predicts complications after DHF in patients older than 50 years who underwent ORIF, providing a valuable tool in surgical risk stratification. In our analysis of 848 patients, 74.1% of patients were female. The increased incidence of this fracture pattern among female patients has previously been described and is consistent with higher prevalence of osteoporosis in females.^8^ Our results demonstrated that a high mFI score was a strong predictor for adverse outcomes after surgical management of DHF: as mFI increased from 0 to 2 or greater, 30-day readmission increased from 3% to 10% and discharge to rehabilitation facility increased from 12% to 32%. Importantly, the risk of complications in patients with mFI score of 2 or greater was significantly higher, most pronounced in the rate of pulmonary complications. This finding is consistent with prior literature involving other metrics of preoperative risk assessment in surgical fields outside of orthopedics, indicating that comorbidity burden may contribute particularly to the development of postoperative pulmonary complications.^15^, ^20^, ^22^ The ASA physical status score, in particular, appears to predict likelihood of postoperative pulmonary complications. Even with protective ventilation practices in place for patients with high ASA scores, mild pulmonary complications such as prolonged oxygen therapy needs after surgery and low-grade atelectasis can have pronounced effects on the perioperative outcome.^15^ Rather than using as a single determinant of postoperative outcomes, we suggest the mFI be considered an additive measure to assess the patient holistically.

We intentionally utilized the 5-item mFI as opposed to the original 11-item mFI for several reasons. First and foremost, one of the primary objectives of this investigation was to increase orthopedic surgeon consideration of patient fragility in the surgical decision-making process for DHF. The 5-item mFI not only takes less time to administer than the 11-item mFI but also is more facile to commit to memorization when speaking with patients. In a busy clinic or in the polytrauma situation, the 5-item mFI is more likely to be applied than the 11-item mFI. Given documented poor ability of orthopedic surgeons to predict fragility on a patient-specific basis,^3^ a simple, effective tool to do so would be of great value. Moreover, the 5-item mFI when compared with the 11-item mFI appears to be equally efficacious.^36^, ^39^

The NSQIP database has several important limitations in consideration of our results. Any large database study has many inherent restrictions in application of data obtained within. Chiefly, statistical significance of large data samples does not always correlate with clinical significance, and large numbers of predictors can often confound results.^25^ Using multivariate regression, we attempted to control for as many confounders as possible. The NSQIP also has limitations specific to this database. Most relevant to this study, the NSQIP is not an orthopedic surgery–specific database and therefore does not include many outcomes that would be of special interest to orthopedic surgeons treating DHF. Tendon rupture, specific neurovascular compromise, radiographic alignment, malunion and nonunion, hardware-specific outcomes, postoperative range of motion, and many other relevant outcomes were not evaluated in this analysis. It is certainly reasonable that many of these outcomes would be adversely affected by higher fragility scores, but further work utilizing other databases is needed to quantify these effect sizes. Additionally, NSQIP database outcomes are only included if they occurred within 30 days after surgery. Thus, there are likely surgery-related complications that are not captured within this time frame. Furthering the limitations of the NSQIP database, only patients undergoing surgery in the hospital setting, inpatient or outpatient, and not patients who obtained their procedure at an ambulatory surgery center are included in this database. Patients with DHF who are able to be discharged initially to follow-up for surgery at an outpatient-only facility are likely to have fewer markers of fragility as opposed to patients who require their surgery during the initial inpatient stay. This introduces a potential source of selection bias into our analysis. Additionally, our study did not stratify outcomes by specific CPT code or ICD diagnostic code. Thus, we forfeit the ability to comment on outcomes of specific fracture patterns as they relate to mFI. Additionally, by choosing not to include the CPT code 24363 for TEA, it is possible that we have failed to include a sampling of periarticular fractures that were ultimately deemed inadequate for ORIF and instead required TEA. Future research should expand upon the utility of mFI in predicting outcomes after TEA for fracture and should aim to stratify by specific fracture subtype.

Finally, this study analyzed only patients who underwent ORIF for treatment of their DHF, leaving out those who underwent nonoperative management. It may be that patients with an mFI >2 who are treated nonoperatively also have medical complications that are not captured in this study.

## Conclusion

Nonoperative versus surgical management of DHF remains a topic of much contention within the orthopedic community. Comorbidity burden may contribute to surgical decision-making, given a significantly higher risk of adverse outcomes after surgical treatment for these fractures in patients with high fragility scores as measured by the mFI. The 5-item mFI appears to be equally efficacious as compared with the 11-item mFI and is a simple tool that orthopedic surgeons can use to help risk-stratify their patients. Future research should elaborate on mFI contribution to orthopedic specific complications not contained within the NSQIP, expand on complications occurring outside of 30 days after the initial procedure, and compare complication profiles for patients who undergo operative versus nonoperative treatment while controlling for mFI scores.

## Disclaimers

*Funding:* No funding was disclosed by the authors.

*Conflicts of interest:* The authors, their immediate family, and any research foundation with which they are affiliated have not received any financial payments or other benefits from any commercial entity related to the subject of this article.
